# Prolonged Estradiol Persistence and Possible Symptom-Biomarker Dissociation During Sequential Estradiol Implant Therapy in a Postmenopausal Woman: A Case Report

**DOI:** 10.7759/cureus.117982

**Published:** 2026-10-15

**Authors:** Loreta C Moraes, Wilmar Accursio, Matheus H Ferreira, Juliana de Oliveira Moreira Gomes Carneiro, Guilherme Renke, Zahid Khan

**Affiliations:** 1 Obstetrics and Gynecology, Federal University of Rio Grande do Sul, Porto Alegre, BRA; 2 Endocrinology and Metabolism, Arthé Clinic, São Paulo, BRA; 3 Physical Education, Physical Therapy and Occupational Therapy, Federal University of Minas Gerais, Belo Horizonte, BRA; 4 Gynecology and Obstetrics, Arthé Clinic, São Paulo, BRA; 5 Endocrinology, Nutrindo Ideais Performance and Nutrition Research Center, Rio de Janeiro, BRA; 6 Cardiology, Barts Heart Centre, Barts Health NHS Trust, London, GBR; 7 Cardiovascular Medicine, William Harvey Research Institute, Queen Mary University of London, London, GBR; 8 Cardiology, Townsville University Hospital, Townsville, AUS

**Keywords:** drug implants, estradiol serum levels, gonadotropins, hormone-replacement therapy, postmenopause

## Abstract

Subcutaneous estradiol implants are a long-acting modality of menopausal hormone therapy whose behavior during sequential implantations remains poorly characterized. We report a 52-year-old woman with natural menopause at the age of 48 years, who was followed for 45 months during five sequential 25 mg estradiol implants combined with gestrinone. Testosterone 50 mg was added from the fourth implantation due to low sexual desire. Estradiol concentrations remained measurable at all assessed post-implantation time points, ranging from 56 to 156 pg/mL. Gonadotropin fluctuations and late recurrence of vasomotor symptoms were observed despite persistent estradiol exposure. Reimplantation decisions were guided by clinical assessment integrated with serial hormonal monitoring rather than fixed intervals. Endometrial protection with dydrogesterone was maintained, and no adverse events were observed or reported. This case illustrates prolonged estradiol persistence, gonadotropin variability, and possible symptom-biomarker dissociation during sequential implant therapy, reinforcing the need for an individualized approach that integrates symptoms, hormone levels, and endometrial assessment.

## Introduction

Subcutaneous estradiol implants provide long-acting menopausal hormone therapy characterized by continuous release and absence of first-pass hepatic metabolism. Although the 25 mg dose has shown clinical efficacy comparable to higher doses [1-3], the pharmacokinetic behavior during sequential implantations remains incompletely characterized, particularly regarding prolonged residual estradiol exposure, stacking, and pseudotachyphylaxis [4-7].

Pharmacokinetic accumulation has been described when reimplantation is performed before complete depletion of the previous implant and may result in sustained supraphysiologic estradiol levels [4-6]. Pseudotachyphylaxis is used here for the reported recurrence of vasomotor symptoms during a declining estradiol trajectory, even when concentrations remain within or above the physiologic range [6,7]. Both terms come from small series and audits, and the supporting evidence is limited. Detailed longitudinal reports integrating serial hormone measurements, gonadotropin dynamics, and clinical outcomes across multiple implant cycles are scarce.

We report a woman followed for 45 months during five sequential 25 mg estradiol implants combined with gestrinone, with testosterone 50 mg added from the fourth implant. The case was notable for prolonged estradiol persistence across cycles and variable gonadotropin concentrations despite measurable estradiol exposure. This report describes the longitudinal hormonal and clinical course and discusses implications for monitoring during sequential implant therapy, following the CAse REport (CARE) guidelines for case reports [8].

## Case presentation

Patient information and clinical findings

A 52-year-old woman with natural menopause at the age of 48 years presented in May 2021 with climacteric symptoms. The contemporaneous record did not itemize these complaints or include a validated symptom score. Baseline estradiol was <11 pg/mL (postmenopausal reference range: ≤20 pg/mL), follicle-stimulating hormone (FSH) 116 IU/L (postmenopausal reference range: 25.8-134.8 IU/L), and luteinizing hormone (LH) 57.3 IU/L (postmenopausal reference range: 19.3-100.6 IU/L), with an atrophic endometrium. She had no relevant comorbidities, chronic medications, or contraindications to hormone therapy. Family history was unremarkable for breast cancer, thromboembolism, or premature cardiovascular disease. She was a non-smoker and physically active. This retrospective case report was approved by the Research Ethics Committee of Hospital Federal de Bonsucesso (HGB), Rio de Janeiro, Brazil, on May 20, 2026, under Opinion No. 8,445,042 and CAAE No. 97223126.3.1001.5253. The study was conducted in accordance with the Declaration of Helsinki.

Timeline

Five subcutaneous implants were placed between March 2022 and October 2025, with laboratory follow-up until December 2025. Intervals between implants were 7, 13, 8, and 15 months. Serial estradiol, FSH, and LH values are shown in Table 1.

**Table 1:** Longitudinal hormonal monitoring and sequential subcutaneous implantations in a postmenopausal woman *Postmenopausal reference ranges (may vary by laboratory assay). Post-implant estradiol levels may exceed the postmenopausal reference range because of therapeutic intent. The reason for each missing sample was not recorded. "-" refers to not measured and not imputed. FSH: follicle-stimulating hormone; LH: luteinizing hormone

Date	FSH (IU/L)	LH (IU/L)	Estradiol (pg/mL)	Clinical context
Postmenopausal reference range*	25.8-134.8	19.3-100.6	≤20	Laboratory-specific reference intervals preferred when available
17 May 2021	116	57.3	<11	Baseline assessment; climacteric symptoms and atrophic endometrium
20 March 2022	-	-	-	First subcutaneous implantation
1 July 2022	21	10.4	70	Fourth month after the first implantation
1 September 2022	21.5	27	71	Sixth month after the first implantation
22 October 2022	-	-	-	Second subcutaneous implantation
22 November 2022	22	13	105	First month after the second implantation
1 January 2023	22.4	13	105	Third month after the second implantation
23 January 2023	17	-	156	Transient estradiol peak
11 February 2023	20	8	88	Fourth month after the second implantation
17 November 2023	-	-	65	Thirteenth month after the second implantation; persistent estradiol exposure
26 November 2023	-	-	-	Third subcutaneous implantation
1 January 2024	17	10	88	First month after the third implantation
6 March 2024	24	14	76	Third month after the third implantation
3 May 2024	31	17	70	Sixth month after the third implantation; increase in gonadotropins
18 June 2024	-	-	70	Seventh month after the third implantation
22 July 2024	-	-	-	Fourth subcutaneous implantation; testosterone added
3 September 2024	12	5	85	First month after the fourth implantation
14 December 2024	13	-	92	Fifth month after the fourth implantation
25 July 2025	71	-	81	Twelfth month after the fourth implantation; marked increase in FSH
13 September 2025	118	-	56	Fourteenth month after the fourth implantation; persistent estradiol with elevated FSH
13 October 2025	-	-	-	Fifth subcutaneous implantation
20 December 2025	17	8.5	138	Second month after the fifth implantation

Serum estradiol, FSH, and LH across the five implant cycles are shown in Figure 1.

**Figure 1:**
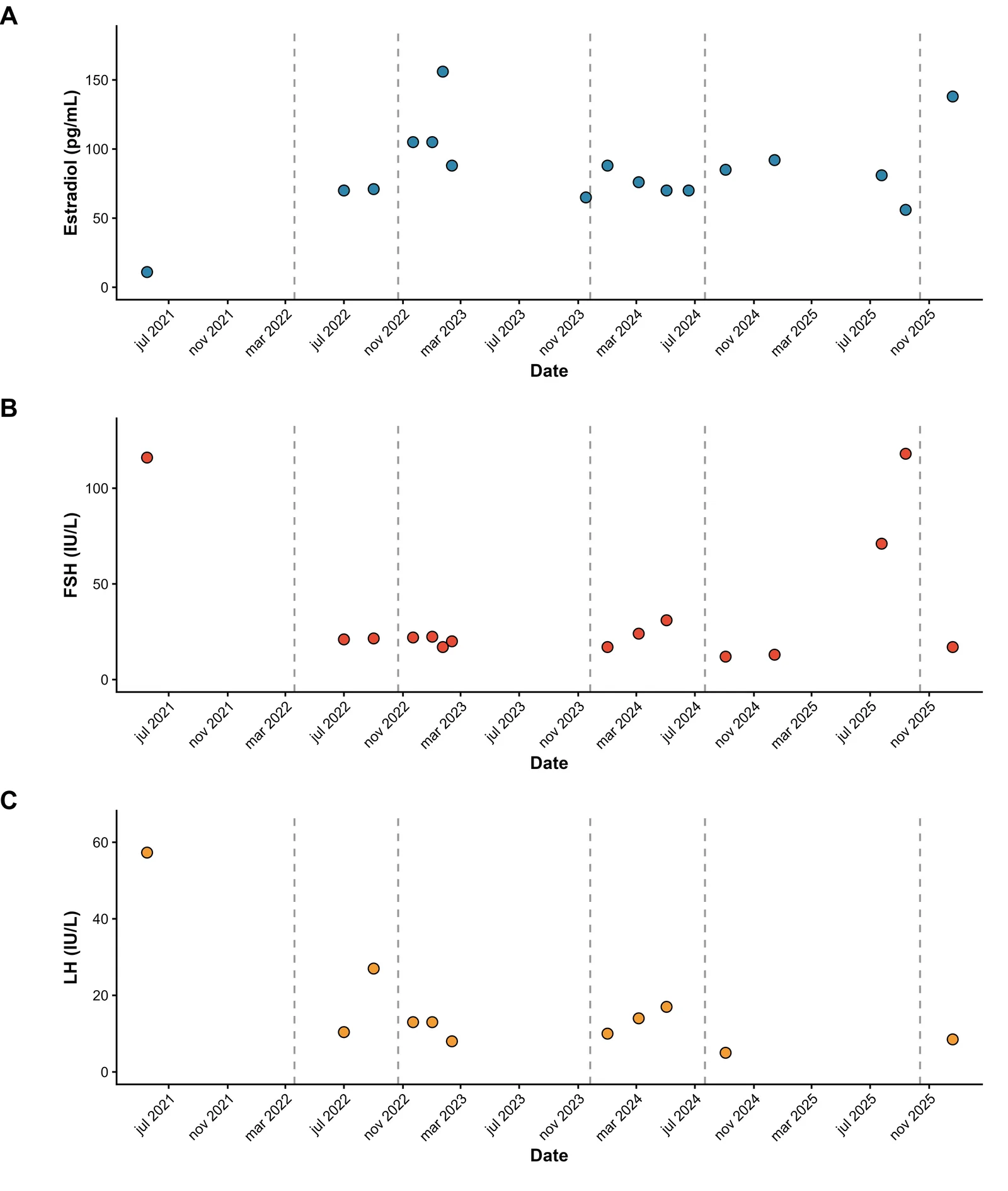
Longitudinal serum (A) estradiol (blue circles), (B) follicle-stimulating hormone (red circles), and (C) luteinizing hormone (orange circles) concentrations during five sequential subcutaneous implant cycles. Only measured values are plotted; gaps are not connected. Vertical dashed lines indicate implantation dates. Missing measurements were not imputed. The figure was created using R version 4.5.1 (R Core Team/R Foundation for Statistical Computing; Vienna, Austria) LH: luteinizing hormone; FSH: follicle-stimulating hormone

Diagnostic assessment

The diagnosis of natural menopause was established by 12 months of amenorrhea at the age of 48 years and confirmed by the baseline hormonal evaluation above. Other causes of climacteric symptoms were excluded. No diagnostic challenges were identified.

Therapeutic intervention

The patient received five sequential subcutaneous implants between March 2022 and October 2025. The first three contained 25 mg estradiol + 35 mg gestrinone; testosterone 50 mg was added from the fourth implant (July 2024) because of low sexual desire. Reimplantation decisions were based on clinical symptoms integrated with serial estradiol, FSH, and LH measurements, rather than fixed intervals. Dydrogesterone 10 mg daily was used for endometrial protection during extended periods beyond six months. The combination of estradiol with gestrinone is a practice used in some Brazilian services for individualized implant therapy.

Follow-up and outcomes

Over 45 months, estradiol remained measurable at every post-implantation assessment (56-156 pg/mL). FSH was generally suppressed after each implantation and rose during the longer intervals; LH rose where it was measured. Initial symptomatic relief was followed by recurrence of vasomotor symptoms in the late phase of two implant cycles, while estradiol remained about 70 pg/mL. These complaints were not itemized and no validated symptom score was used. Dydrogesterone was continued across the extended intervals. No adverse events or implant-related complications were observed.

Symptom severity was not graded, reasons for missing hormone measurements were not recorded, and no serial endometrial ultrasound or biopsy was documented.

## Discussion

This case differs from the classic descriptions of implant tachyphylaxis. Garnett et al. reported return of estrogen-deficiency symptoms before estradiol had fallen to postmenopausal values, with supraphysiologic levels (>1750 pmol/L, about 477 pg/mL) in about 3% of implant users [6]. Buckler et al. deferred reimplantation when estradiol remained above 400 pmol/L (about 109 pg/mL) and found no progressive accumulation, with intervals lengthening to about 12 months [4]. In the present patient, five 25 mg implants over 45 months produced intervals of 7, 13, 8, and 15 months and estradiol of 56-156 pg/mL, without progressive accumulation. Residual release, not accumulation, accounts for the estradiol of 65 pg/mL still present 13 months after the second implant. Prolonged endometrial stimulation after implants is well documented [5], and the long duration of action in non-hysterectomized women supports continued progestogen during these intervals [7].

The clinically relevant finding is symptom-biomarker dissociation at moderate, not supraphysiologic, concentrations. Vasomotor symptoms returned in two late cycles while estradiol was still about 70 pg/mL, consistent with a response to relative decline rather than to absolute deficiency [6,7]. These complaints were not itemized, and no validated symptom score was used; thus, the dissociation is anchored to the estradiol trajectory. Over the same intervals, FSH rose despite relatively stable estradiol, and LH rose where it was measured. In this single case, that pattern is compatible with a non-linear hypothalamic-pituitary response, but it does not establish FSH as a monitoring marker. This case raises the possibility that symptoms or a fixed calendar may miss residual release. It does not establish that estradiol, or gonadotropins, should determine the timing of reimplantation. A 70 pg/mL value was discussed locally as a conservative point at which to review, rather than insert, an implant; it was not applied as a validated cut-off and is more conservative than Buckler’s 109 pg/mL threshold [4].

A transient peak of 156 pg/mL in the third month of the second implant, falling to 88 pg/mL the next month, may reflect late pellet fragmentation, although biological and analytical variations cannot be excluded [9]. Because this is a single case, these observations are hypothesis-generating. Prospective series with systematic symptom scores are needed to test whether gonadotropin escape adds useful information to estradiol when timing sequential 25 mg implants.

## Conclusions

Sequential 25 mg estradiol implants released hormone for 13-15 months in this patient, without progressive accumulation, and vasomotor symptoms returned while estradiol was still measurable. Reimplantation was timed based on symptoms and serial hormone levels, rather than from a fixed interval, and progestogen was continued across the long intervals.

These single-case observations are hypothesis-generating and do not define a monitoring cut-off. Prospective series with systematic symptom scores are needed before these findings can guide practice.
